# Glucose-independent segmental phase angles from multi-frequency bioimpedance analysis to discriminate diabetes mellitus

**DOI:** 10.1038/s41598-017-18913-7

**Published:** 2018-01-12

**Authors:** Min-Ho Jun, Soochan Kim, Boncho Ku, JungHee Cho, Kahye Kim, Ho-Ryong Yoo, Jaeuk U. Kim

**Affiliations:** 1 KM Fundamental Research Division, Korea Institute of Oriental Medicine (KIOM), 1672 Yuseongdaero, Yuseong-gu, Daejeon Republic of Korea; 2 Department of Electrical and Electronic Engineering & Institute for IT Convergence, Hankyong National University, 327 Jungang-no, Anseong-si, Gyeonggi-do Republic of Korea; 3 Internal Medicine of Cardiovascular, Daejeon Oriental Hospital of Oriental Medical College of Daejeon University, 22-5 Daehung-dong, Jung-gu, Daejeon 301-724 Republic of Korea

## Abstract

We investigated segmental phase angles (PAs) in the four limbs using a multi-frequency bioimpedance analysis (MF-BIA) technique for noninvasively diagnosing diabetes mellitus. We conducted a meal tolerance test (MTT) for 45 diabetic and 45 control subjects stratified by age, sex and body mass index (BMI). HbA1c and the waist-to-hip-circumference ratio (WHR) were measured before meal intake, and we measured the glucose levels and MF-BIA PAs 5 times for 2 hours after meal intake. We employed a t-test to examine the statistical significance and the area under the curve (AUC) of the receiver operating characteristics (ROC) to test the classification accuracy using segmental PAs at 5, 50, and 250 kHz. Segmental PAs were independent of the HbA1c or glucose levels, or their changes caused by the MTT. However, the segmental PAs were good indicators for noninvasively screening diabetes In particular, leg PAs in females and arm PAs in males showed best classification accuracy (AUC = 0.827 for males, AUC = 0.845 for females). Lastly, we introduced the PA at maximum reactance (PAmax), which is independent of measurement frequencies and can be obtained from any MF-BIA device using a Cole-Cole model, thus showing potential as a useful biomarker for diabetes.

## Introduction

The bioelectrical impedance analysis, or bioimpedance analysis (BIA), which is used to diagnose and monitor pathologies of the human body, was developed in the early 1960 s, and it has been recognized as a safe, rapid, reliable, easy, and cost-effective technique. BIA is a measurement technique based on the electrophysiological characteristics of the dielectric and conductive properties of human tissues. BIA has been widely used to measure body composition^1–3^. Recently, many studies have reported the possibility of using BIA to measure health status indicators and/or clinical outcomes in clinical populations using raw bioimpedance parameters, such as reactance, resistance, and phase angle (PA)^4–13^. In particular, PA has been extensively investigated as an important index for monitoring and screening various diseases and conditions, such as mortality, nutrition status, diabetes, hemodialysis, chronic heart failure, and liver cirrhosis, etc.^14–19^.

According to previous studies, PA is an indicator of the distribution of the intra- and extracellular water of cells^20^ and an indicator of the amount of electrical charge that cell membranes can hold because the PA is related to the total cell membrane mass^19^. Based on these studies, the PA seems to be closely related to cell activity or the metabolism of the human body. Actually, some studies have reported the diagnostic utility of the PA in people with diabetes mellitus^20–24^. Previously, bioimpedance studies related to diabetes mellitus analyzed body composition as a risk factor for diabetes mellitus^25–28^. More recently, raw bioimpedance parameters such as the PA, resistance and reactance have been directly analyzed at different frequencies in people with diabetes mellitus. Despite these achievements, however, a deeper understanding will be required before more practical applications of MF-BIA and its PAs can be developed for diagnosing and monitoring diabetes. One of these research questions will be whether the changes in the PAs follow real-time glucose changes or glycated hemoglobin level (HbA1c) values. Another such question may be whether the PAs obtained at different body parts show different behaviors in classifying diabetic patients. According to previous studies, local lesions among diabetic complications, such as diabetic foot, diabetic neuropathy, and peripheral vascular disease, have been well documented^29–32^. It is necessary to determine whether these local lesions are reflected in the segmental PAs or bioimpedance signals.

In this study, we investigated the statistical differences between the segmental PAs (right arm, RA; left arm, LA; right leg, RL; left leg, LL; and trunk, TR) obtained by multi-frequency bioimpedance analysis (MF-BIA) in patients with diabetes mellitus, changes in the segmental PAs after a meal tolerance test (MTT), and the feasibility of noninvasively screening patients with diabetes mellitus. For this purpose, we conducted a MTT and measured segmental PAs with glucose and HbA1c levels for diabetic patients and age-matched, sex-matched, and body mass index (BMI)-matched healthy controls. For the analysis, we first tested for the statistical differences in the 5 segmental PAs and the influence of the MTT between the patients with diabetes mellitus and the healthy controls. Next, we introduced the PA at maximum reactance, or “PAmax”, which can be obtained by any MF-BIA method and is independent of the measurement frequencies of BIA devices. Finally, we examined the classification accuracy of the segmental PAs and the PAmax using the area under the curve (AUC) of a receiver operating characteristic (ROC) curve for diabetes mellitus.

## Results

### Patient demographic and clinical characteristics

The mean ± SD age of the male patients with diabetes mellitus (n = 26) and controls (n = 26) were 60.19 ± 9.22 years and 57.69 ± 8.85 years, respectively, and the ages of the female patients with diabetes mellitus (n = 19) and controls (n = 19) were 61.53 ± 7.46 years and 58.58 ± 8.92 years, respectively. The demographic data and laboratory tests for the patients with diabetes mellitus and the control subjects are summarized in Table 1. The age, weight, height and BMI of the diabetes and control groups were not significantly different, except for the BMI of females (P = 0.041). The waist and hip circumferences, waist-to-hip-circumference ratio (WHR), plasma glucose level, FPG level, HbA1c, and skin humidity in the diabetic patients and control subjects are shown in Table 1. The differences in plasma glucose levels, FPG levels, and HbA1c levels were statistically significant between the diabetes and controls groups, which is well known clinically. The fasting glucose levels of the participants in the control group were 102.56 ± 11.05 mg/dl (5.69 ± 0.61 mmol/l) and 135.78 ± 30.41 mg/dl (7.54 ± 1.69 mmol/l) in the diabetes group, and the HbA1c was 5.65 ± 0.47% (38.25 ± 5.14 mmol/mol) in the control group and 6.78 ± 0.82% (50.61 ± 8.96 mmol/mol) in the diabetes group. In addition, medical treatment status, medications, and diabetes duration are shown in Table 2. Among the 45 diabetic participants, 40 subjects were receiving treatment for diabetes and 5 were treatment-naïve before enrollment in this study. Of the 40 subjects receiving treatment, 39 were taking oral antidiabetic drugs and one was taking antihypertensive drugs. None of the participants were on insulin therapy.

**Table 1:** Demographic and laboratory data for the diabetic patients and controls.

Variable	Males (n = 52)		Statistics	Females (n = 38)		Statistics
	Diabetics (n = 26) Mean ± SD	Controls (n = 26) Mean ± SD	*P-values*	Diabetics (n = 19) Mean ± SD	Controls (n = 19) Mean ± SD	*P-values*
Age (years)	60.19 ± 9.22	57.69 ± 8.85	0.323	61.53 ± 7.46	58.58 ± 8.92	0.277
Weight (kg)	68.74 ± 9.66	70.48 ± 10.75	0.542	59.93 ± 8.80	55.49 ± 7.57	0.104
Height (cm)	167.29 ± 7.30	167.23 ± 4.16	0.972	152.68 ± 5.97	152.99 ± 6.33	0.875
BMI (kg/m^2^)	24.56 ± 3.03	25.17 ± 3.47	0.507	25.66 ± 3.14	23.68 ± 2.60	0.041*
Waist circumference (cm)	90.63 ± 7.90	89.5 ± 8.73	0.625	87.32 ± 9.41	81.63 ± 6.20	0.034*
Hip circumference (cm)	97.96 ± 5.77	99.69 ± 6.87	0.330	94.87 ± 5.22	93.74 ± 4.69	0.487
WHR	0.92 ± 0.05	0.90 ± 0.04	0.024*	0.92 ± 0.08	0.87 ± 0.06	0.037*
Glucose level (mg/dl)	208.71 ± 57.69	135.83 ± 44.13	<0.001***	192.28 ± 61.57	125.85 ± 28.45	<0.001***
Fasting Glucose (mg/dl)	148.04 ± 29.87	105.88 ± 12.25	<0.001***	133.68 ± 35.91	99.37 ± 7.73	<0.001***
HbA1c (%)	6.96 ± 0.82	5.56 ± 0.51	<0.001***	6.53 ± 0.78	5.77 ± 0.40	<0.001***
Skin Humidity (%)	32.93 ± 3.58	32.56 ± 3.84	0.417	34.20 ± 7.36	32.76 ± 2.22	0.070

P-values: *<0.05, **<0.01, ***<0.001; BMI: body mass index, WHR: waist to hip ratio, Glucose level: average level of 5 measurements in 2 hours, Fasting Glucose: glucose level before meal intake, HbA1c: glycated hemoglobin.

**Table 2:** Treatment status, medications, and diabetes duration among the enrolled diabetes patients.

Health information		Total	Males	Females
Medical status	No. of diabetic patients (n)	45	26	19
	Treated (n)	40	23	17
	Un-treated (n)	5	3	2
Medication	Oral antidiabetic drug (n)	39	23	16 + 1 (antihyper. drug)
	Antiarteriosclerotic drug (n)	30	14	16
	Antihypertensive drug (n)	25	14	11
	Other cardiovascular drugs (n)	13	6	7
Regimen	Only antidiabetic drug (n)	11	9	2
	2 kinds of drugs (n)	19	10	9
	More than 3 kinds of drugs (n)	9	4	5
Duration (years)		7.91 ± 7.40	8.46 ± 8.18	7.16 ± 7.14

The means, SDs, and P-values obtained from the t-tests and the adjusted p-values for the averaged segmental PAs and the averaged whole-body PAs of the diabetic patients and controls are shown in Table 3. There are statistically significant differences in the segmental PAs between the diabetic patients and controls. In the male group, the PAs at 5 kHz-RA, 50 kHz-RA, 50 kHz-LA, 50 kHz-RL, 250 kHz-RA, 250 kHz-LA, 250 kHz-RL, and 250 kHz-LL of the diabetic and control groups were significantly different. In the female group, the PAs at 5 kHz-RL, 5 kHz-LL, 50 kHz-RL, 50 kHz-LL, 250 kHz-RA, 250 kHz-LA, 250 kHz-RL, and 250 kHz-LL of the two groups were significantly different. All segmental PAs of 250 kHz of the two groups, except for those of the TR, were significantly different. In the cases of 5 kHz and 50 kHz, the segmental PAs that showed significant differences between the two groups were only for parts of the arms in the male group and for parts of the legs in the female group. Additionally, to verify the statistical significances, we repeated the analysis based on the adjusted P-values for multiple comparisons. The P-value adjustments in this clinical trial were performed to compensate for a possible increased risk in committing Type I errors when multiple outcome measures were used. When comparing the non-adjusted *P*-values with the adjusted p-values, the statistical significances were similar to the results based on the non-adjusted p-values, and the number of P-values less than 0.05 was identical to the number of adjusted p-values with statistical significance. Whole-body PAs were obtained using a serial connection model with the impedance values of the right arm, trunk, and right leg^22^. The whole-body PAs were significantly different between genders, but the significance levels were lower than those of the best segmental PAs (arm PAs for males and leg PAs for females). In addition, there were significant differences between the right and left sides and between the arms and legs, but there was no significant difference between the right and left legs as shown in Table A1 of the Appendix.

**Table 3:** T-test results of averaged segmental phase angles and averaged whole body phase angles for diabetic patients and controls.

Variable		Male (n = 52)		Statistics		Female (n = 38)		Statistics	
		Diabetes (n = 26)	Controls (n = 26)	*P-values*	*Adj. P*	Diabetes (n = 19)	Controls (n = 19)	*P-values*	*Adj. P*
Segmental PA (degrees)	5 kHz-RA	2.55 ± 0.35	2.82 ± 0.45	0.009**	0.023	2.25 ± 0.30	2.63 ± 1.31	0.172	0.221
	5 kHz-LA	2.35 ± 0.38	2.57 ± 0.40	0.050	0.082	2.23 ± 1.51	2.16 ± 0.28	0.393	0.417
	5 kHz-TR	3.52 ± 1.60	3.27 ± 2.15	0.478	0.491	3.18 ± 1.12	3.26 ± 1.16	0.282	0.307
	5 kHz-RL	2.99 ± 0.78	3.25 ± 0.66	0.175	0.221	2.24 ± 0.38	2.69 ± 0.49	0.002**	0.011
	5 kHz-LL	2.85 ± 0.66	3.09 ± 0.70	0.184	0.221	2.29 ± 0.61	2.63 ± 0.49	0.016*	0.029
	50 kHz-RA	5.83 ± 0.82	6.49 ± 0.85	0.002**	0.011	5.36 ± 0.57	5.71 ± 0.79	0.181	0.221
	50 kHz-LA	5.48 ± 0.58	6.08 ± 0.81	0.002**	0.011	4.92 ± 0.50	5.20 ± 0.40	0.060	0.092
	50 kHz-TR	6.67 ± 2.63	7.27 ± 3.31	0.061	0.092	5.75 ± 2.01	6.26 ± 1.84	0.232	0.261
	50 kHz-RL	6.84 ± 0.96	7.52 ± 0.97	0.014*	0.028	5.60 ± 0.86	6.64 ± 1.00	0.002**	0.011
	50 kHz-LL	6.56 ± 0.92	7.13 ± 1.36	0.081	0.113	5.60 ± 0.89	6.44 ± 0.95	0.008**	0.023
	250 kHz-RA	5.78 ± 0.64	6.55 ± 1.57	0.002**	0.011	5.45 ± 0.61	5.90 ± 0.80	0.021*	0.036
	250 kHz-LA	5.52 ± 0.59	6.27 ± 1.52	<0.001***	0.010	5.13 ± 0.48	5.54 ± 0.60	0.009**	0.023
	250 kHz-TR	8.43 ± 2.50	8.99 ± 3.59	0.631	0.631	6.89 ± 2.50	7.67 ± 2.17	0.199	0.231
	250 kHz-RL	3.59 ± 0.54	4.08 ± 0.81	0.010*	0.023	3.28 ± 0.58	4.00 ± 0.62	<0.001***	0.005
	250 kHz-LL	3.53 ± 0.56	4.08 ± 0.86	0.006**	0.020	3.33 ± 0.71	3.86 ± 0.61	0.015*	0.028
Whole body	PA_5 kHz	2.77 ± 0.48	3.01 ± 0.41	0.066	0.095	2.30 ± 0.25	2.53 ± 0.27	0.011*	0.025
	PA_50 kHz	6.22 ± 0.72	6.89 ± 0.76	0.002**	0.011	5.46 ± 0.58	6.06 ± 0.65	0.005**	0.017
	PA_250 kHz	5.35 ± 0.49	6.01 ± 1.18	0.012*	0.025	4.94 ± 0.54	5.47 ± 0.49	0.003**	0.012

P-values: *<0.05, **<0.01, ***<0.001; RA: right arm, LA: left arm, TR: trunk, RL: right leg, LL: left leg, PA: phase angle; Adj. P: represents adjusted P-values based on Benjamini-Hochberg’s procedure.

The relationship between segmental PAs and disease duration is shown in Table A2 of the Appendix. The correlation between PA and disease duration was most prominent for the PAs at 50 kHz, and males showed the stronger correlation, with |r| ≤ 0.451 at 50 kHz-RA compared to |r| ≤ 0.580 at 50 kHz-RL among females. Interestingly, the correlation coefficient (r_Pearson_) of segmental PAs at 50 kHz in the arms of males and in the legs of females was higher than those of the other segments. In addition, body composition data obtained by the bioimpedance device were analyzed to screen diabetic patients and controls as shown in Table S2 of the Appendix. No differences were observed between the diabetic patients and the controls in muscle mass, skeletal muscle mass, and segmental muscle mass, and weak correlations between PAs and muscle mass (|r| ≤ 0.50) and skeletal muscle mass (|r| ≤ 0.54) are shown in Table A3 of the Appendix.

The correlation between the segmental PAs and BMI was analyzed because 8 of the 15 segmental PAs were significantly different between the diabetic patients and control subjects in the female group. The correlations between the PAs and BMI were considered to be weakly negative correlations because the maximum correlation coefficient (*r*) was −0.384 at 250 kHz-LA. This weak correlation means that the segmental PAs are not directly reflected as indicators of BMI. There were weak correlations between the glycated hemoglobin level, fasting plasma glucose level, BMI or WHR, and segmental PAs, as the values of the maximum correlation coefficient between the PAs and HbA1c, fasting glucose levels, BMI, and WHR were −0.327, −0.307, 0.322, and −0.353, respectively, as shown in Table 4. Therefore, the segmental PA is not a direct indicator of the HbA1c, fasting glucose level, BMI, or WHR, but it could be an independent biomarker of diabetes. The correlations between the impedances and skin humidity were analyzed to determine the influence of the electrical resistance and skin humidity. However, we found little evidence of a correlation between them in the analysis results (r = 0.07). The PAs of the TR are excluded from the correlation analysis because of their large SDs.

**Table 4:** Correlations between segmental PAs and HbA1c, fasting glucose, BMI, and WHR.

Variables	Phase angle vs. HbA1c		Phase angle vs. glucose		Phase angle vs. BMI		Phase angle vs. WHR	
	r_Pearson_	*P-values*	r_Pearson_	*P-values*	r_Pearson_	*P-values*	r_Pearson_	*P-values*
5 kHz-RA	−0.301	0.004**	−0.189	0.077	0.179	0.096	−0.024	0.826
5 kHz-LA	−0.219	0.040*	−0.135	0.209	0.188	0.080	−0.040	0.709
5 kHz-RL	−0.280	0.008**	−0.153	0.154	0.332	0.002**	−0.120	0.267
5 kHz-LL	−0.217	0.041*	−0.126	0.240	0.288	0.006**	−0.134	0.210
50 kHz-RA	−0.270	0.011*	−0.254	0.017*	0.102	0.342	−0.121	0.262
50 kHz-LA	−0.246	0.021*	−0.213	0.046*	0.128	0.234	−0.119	0.270
50 kHz-RL	−0.278	0.008**	−0.194	0.069	0.154	0.151	−0.250	0.018*
50 kHz-LL	−0.234	0.027*	−0.164	0.124	0.087	0.418	−0.296	0.005**
250 kHz-RA	−0.269	0.011*	−0.254	0.016*	−0.116	0.280	−0.181	0.090
250 kHz-LA	−0.244	0.021*	−0.222	0.037*	−0.154	0.148	−0.182	0.087
250 kHz-RL	−0.327	0.002*	−0.307	0.004*	0.034	0.750	−0.353	<0.001***
250 kHz-LL	−0.267	0.011*	−0.267	0.012*	−0.046	0.667	−0.331	0.02

^*^r_Pearson_: Pearson’s correlation coefficient.

### Segmental phase angles with the meal tolerance test (MTT)

The segmental PAs and blood glucose levels of all of the subjects were measured for 2 hours, starting before meal intake and then every 30 minutes after meal intake. The analysis results between diabetes and controls were slightly different according to the measurement time but there was no significant change of segmental PAs with measurement time. The mean ± SD of the segmental PAs and p-values, as the results of t-test between the diabetes and control groups were shown in Table A4 in the Appendix.

**Figure 1:**
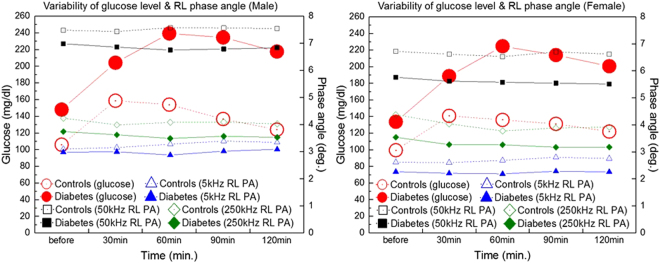
Plasma glucose levels (: diabetics, : controls; left axis) and phase angles (5 kHz-RL (: diabetics, : controls), 50 kHz-RL (■: diabetics, □: controls), 250 kHz-RL (: diabetics, : controls), right axis) of controls and diabetic patients among males and females at various measurement times; measurement results before meal intake (MI) and every 30 minutes after meal intake for 2 hours.

The plasma glucose levels and PAs at 5 kHz-RL, 50 kHz-RL, and 250 kHz-RL, with respect to the measurement time, for the control and diabetes groups are presented in Fig. 1. The plasma glucose levels rapidly increased after meal intake and then gradually decreased with time. The mean FPG level of the control group was 103.13 ± 10.97 mg/dl, and that of the diabetes group was 141.98 ± 32.95 mg/dl. The mean glucose levels in the control and diabetes groups increased to 146.36 ± 47.71 mg/dl and 233.07 ± 57.51 mg/dl, respectively, an hour after meal intake and gradually decreased to 123.09 ± 27.33 mg/dl and 210.38 ± 58.80 mg/dl, respectively, two hours after meal intake. However, the segmental PAs in the RL did not show such significant variations over time. Similarly, the PAs in other segmental areas showed no significant variations between the measurements of time. These results suggest that segmental PAs do not reflect the glucose level or its changes. Therefore, segmental PAs could be considered a new biomarker for classifying diabetics from healthy people, independent of the conventional criteria based on blood glucose levels.

In the results of the analysis, the PAs of the arm (RA and LA) in the male group and the PAs of the leg (RL and LL) in the female group were found to better classify people with diabetes. In addition, measuring the PAs after a specific period of time after meal intake could improve the classification accuracy of diabetic patients. Particularly, the PA in the LA at 250 kHz 120 minutes after meal intake showed the greatest statistical differences in the males, while the PA in the RL at 250 kHz 30 minutes after meal intake indicated the greatest difference in the females.

### Phase angle at the maximum reactance value (PAmax)

**Figure 2:**
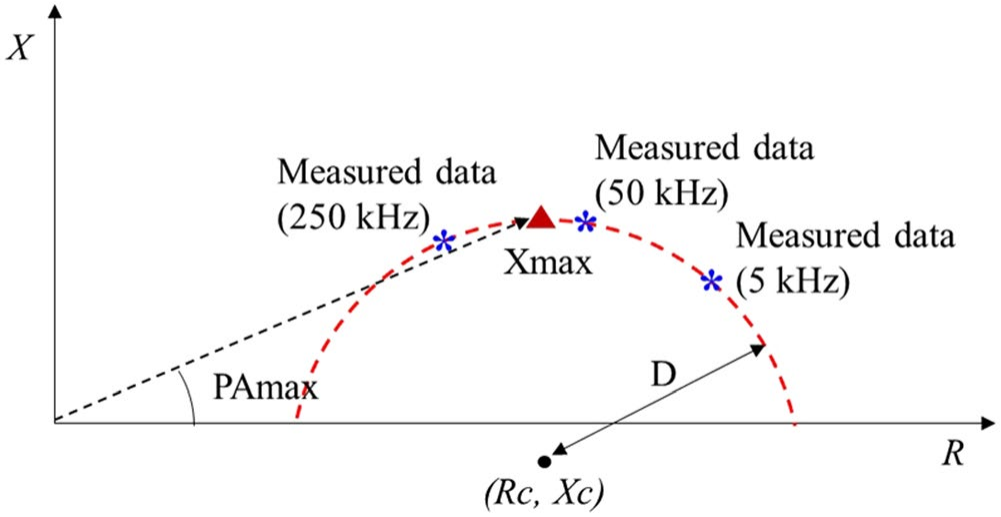
Circle fitting with the measured impedance values at three different frequencies (5 kHz, 50 kHz, and 250 kHz), and the definition of PAmax.

For further analysis, we introduced “PAmax”, which we define as the PA at the maximum reactance value. The advantage of this variable is that PAmax can be easily derived from any MF-BIA device using a circular equation in the Cole-Cole model, and it is independent of the various frequencies adopted by diverse types of BIA devices. In addition, PAmax is considered the best indicator of electrical resistance and reactance of the body because differences at maximum reactance are the most pronounced^33^. PAs are directly related to cell membranes, and PAs can be calculated as the arc-tangent of the ratio of reactance to resistance. Since the impedance signal measured from the human body is similar to that produced by the Cole-Cole model, a circular equation can be derived by fitting a circle from three points that are measured at different frequencies, 5 kHz, 50 kHz, and 250 kHz, in the case of used MF-BIA equipment (InBody S10, InBody, Korea). The fitted circular equation is as follows:

1 $${({x}-{Rc})}^{2}+{({y}-{Xc})}^{2}={{D}}^{2},$$

2 $${\rm{PAmax}}=\arctan (\frac{{Xmax}}{{Rc}}),$$

where Rc and Xc are the x and y coordinates of the center of the circle, respectively, D is its radius, and PAmax is the PA of Xmax, which is defined by the angle between the coordinates (Rc, Xmax) and (Rc, 0), as shown in Fig. 2. Xmax is the maximum reactance calculated using the circular equation (equation (1)). The means and SDs of the calculated Rc, Xmax and PAmax values are shown in Table 5. We excluded PAmax from the analysis if the calculated Rc was not located between the resistances of 5 kHz and 250 kHz. The PAs in the TR were excluded from the analysis because fitting the circle with the measured TR data was difficult. The Rc, Xmax and PAmax were analyzed by a t-test, and the Xmax and PAmax showed significant differences between the diabetes and control groups. As shown in Table 5, the PAmax-RA and PAmax-LA in males showed notably lower *P*-values than those of any of the measured PAs, which indicates that PAmax is a potential biomarker that can replace existing multi-frequency analysis variables.

**Table 5:** T-test results for calculated Rc, Xmax, and PAmax values between diabetic patients and controls.

Variable	Males (n = 52)		Statistics	Females (n = 38)		Statistics
	Diabetics (n = 26)	Controls (n = 26)	*P-values*	Diabetics (n = 19)	Controls (n = 19)	*P-values*
Rc-RA (Ω)	262.21 ± 20.26	262.65 ± 35.43	0.957	299.59 ± 29.53	319.69 ± 38.25	0.078
Rc-LA (Ω)	267.11 ± 23.08	266.17 ± 35.74	0.911	298.56 ± 33.39	324.66 ± 36.96	0.028*
Rc-RL (Ω)	142.86 ± 12.87	149.86 ± 17.32	0.104	158.84 ± 17.55	171.22 ± 24.03	0.078
Rc-LL (Ω)	147.80 ± 12.98	150.92 ± 17.97	0.477	160.66 ± 18.19	174.42 ± 24.01	0.054
Xmax-RA (Ω)	28.03 ± 3.10	31.44 ± 4.27	0.002**	29.74 ± 3.99	34.05 ± 5.88	0.012**
Xmax-LA (Ω)	27.00 ± 2.54	30.29 ± 4.36	0.002**	27.95 ± 3.77	32.00 ± 4.27	0.004**
Xmax-RL (Ω)	17.00 ± 2.45	19.53 ± 2.91	0.001**	15.58 ± 3.39	19.87 ± 4.58	0.002**
Xmax-LL (Ω)	16.84 ± 2.34	18.54 ± 3.43	0.041*	15.66 ± 3.53	19.64 ± 4.57	0.005**
PAmax-RA (deg.)	6.11 ± 0.52	6.67 ± 0.46	<0.001***	5.67 ± 0.52	5.96 ± 0.46	0.080
PAmax-LA (deg.)	5.78 ± 0.45	6.28 ± 0.34	<0.001***	5.29 ± 0.46	5.61 ± 0.38	0.027*
PAmax-RL (deg.)	6.81 ± 0.95	7.45 ± 0.94	0.017*	5.57 ± 0.85	6.59 ± 0.96	0.001**
PAmax-LL (deg.)	6.52 ± 0.90	7.06 ± 1.33	0.092	5.56 ± 0.89	6.39 ± 0.92	0.008**

Rc and Xc: the center of the fitted circle, Xmax: the maximum reactance, PAmax: the angle between the radial direction at Xmax and the horizontal axis.

### Classification accuracy of multiple phase angles

**Figure 3:**
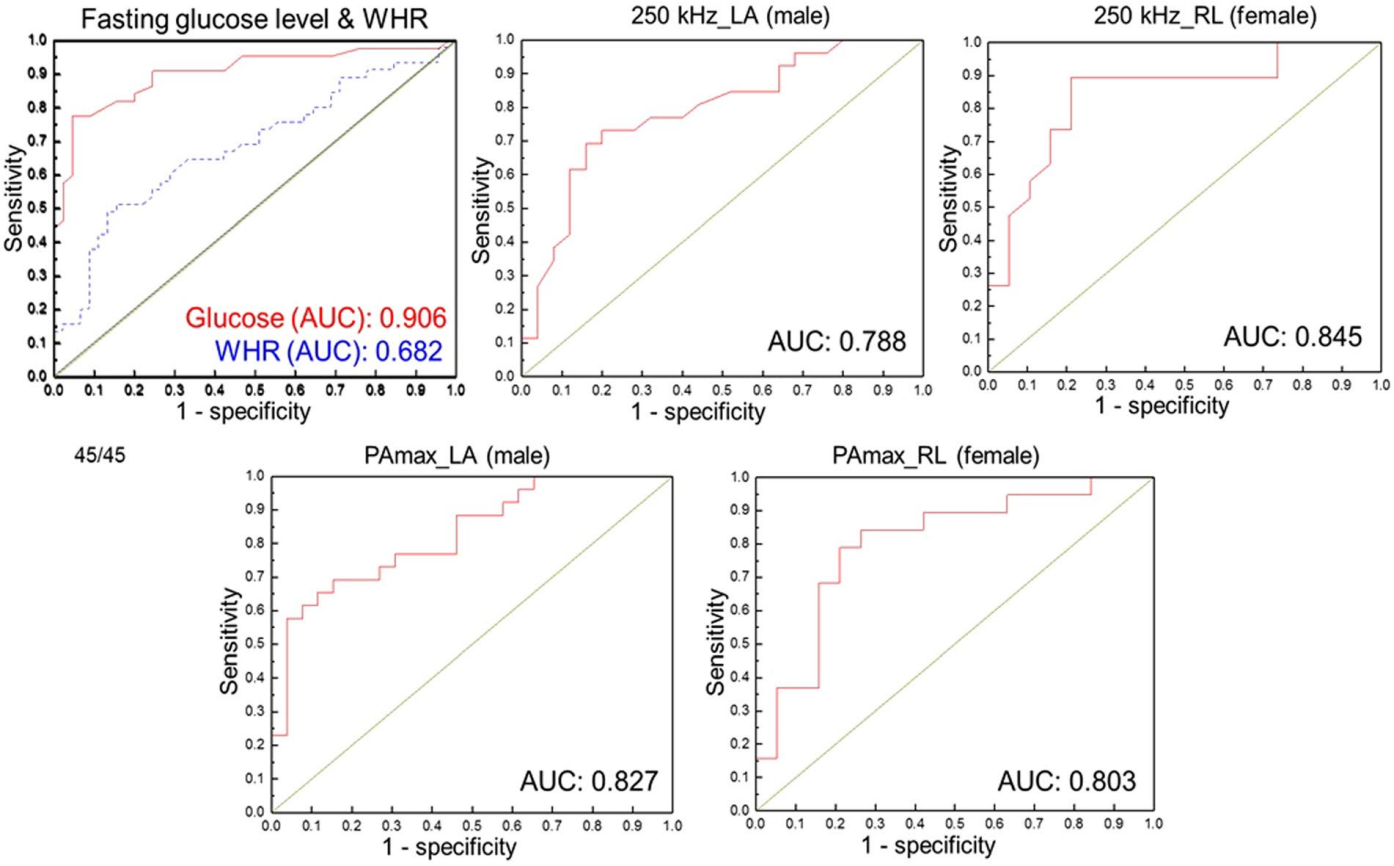
ROC curve and area under the ROC curve (AUC) values of fasting plasma glucose level (red solid line), WHR (blue dot line), left arm phase angle at 250 kHz in males, right leg phase angle at 250 kHz in females, left arm PAmax in males, and right leg PAmax in females.

The fasting plasma glucose level, WHR, and segmental PAs were compared between the groups using the AUC of ROC to determine the PA’s potential for diagnosing diabetes, and the results are shown in Fig. 3. Segmental PAs were compared with the fasting glucose level and WHR because the fasting glucose level is the gold standard, and WHR is also a well-known indicator of diabetes. The AUC values of fasting glucose level, WHR, the PA at 250 kHz-LA in males, the PA at 250 kHz-RL in females, PAmax-LA in males, and PAmax-RL in females are 0.906 (95% CI, 0.84 to 0.97), 0.682 (95% CI, 0.57 to 0.79), 0.788 (95% CI, 0.66 to 0.91), 0.845 (95% CI, 0.72 to 0.97), 0.827 (95% CI, 0.72 to 0.94), and 0.803 (95% CI, 0.66 to 0.95), respectively. The largest AUC values were obtained from the calculated PAmax-LA in males and the measured PA of the RL at 250 kHz in females. Interestingly, a larger AUC value in males was obtained from the calculated PAmax variation than from the measured PAs. Therefore, classifying diabetes by the maximum reactance or PAmax derived from the measured data using multi-frequency bioimpedance would be more effective.

## Discussion

Recent publications have reported some achievements in classifying diabetes from raw bioimpedance data. However, no studies have revealed a clear mechanism underlying the significant differences observed in the bioimpedance data between the diabetes and healthy control groups thus far. Some studies have introduced the mechanism of changes in the electrical characteristics for diabetic patients. Others have shown that the problems with nerve endings^34^ and microcirculation^30^ were causes of diabetic foot ulcers. Hence, a change in the electrical properties of the foot skin might be expected for diabetic patients. Brem and Tomic-Canic^35^ showed electrical differences in hallux bioimpedance of control and diabetic subjects. The hallux is a representative human body part where the skin becomes damaged due to diabetic foot disease, and this is usually manifested as microscopic ulcers. Pillon *et al*.^24^ showed that people with diabetes have a reduced ability to exchange potassium in their bodies due to a decreased active cell mass^25^. Some related papers have claimed that total body potassium is an acceptable indicator of body cell mass^36^ and showed that the PA at 100 kHz was strongly correlated with total body potassium (r = 0.70) in people with diabetes^16^. These results suggest that smaller PAs in people with diabetes indicate cell death or a decreased cell mass, but the larger PAs in people without diabetes indicate an increased cell mass and larger quantities of intact cell membranes^19^. In addition, PAs might also show the nutrition status of patients because the PA at 50 kHz was positively correlated with serum markers of the nutrition^21^. Therefore, the smaller PAs in people with diabetes could be construed as indicators for the catabolic state in people with diabetes^16^.

Di Mauro *et al*.^25^ reported a significant relationship between HbA1c level and exchangeable body potassium, and diabetic patients were found to have a lower quantity of extracellular water and exchangeable potassium in the body. Buscemi *et al*.^20^ observed a negative correlation between fasting plasma glucose level and the PA at 50 kHz and the PA was inversely related to the extracellular to intracellular water ratio because hyperglycemia in patients may induce an osmotic effect that results in a higher resistance value. A higher resistance value results in a smaller PA because the PA is a function of the ratio of reactance to resistance. Some papers have reported that the extracellular water space might be reduced in people with diabetes as a result of hyperglycemia-related osmotic diuresis^37^ and alterations in plasma osmolality^25^. These are explanations of the mechanism underlying the smaller PAs observed in diabetic patients. Based on these results, reduced PAs can be considered an independent indicator of pathologic status in people with diabetes. On the other hand, low PAs have previously been reported in patients with other types of diseases, such as chronic obstructive pulmonary disease (COPD)^38^, HIV^39^, cancer^40^, and anorexia nervosa^41^, and following cardiac surgery^42^. Therefore, a stringent study design will help clarify the potential contribution of PAs and other features of bioimpedance to distinguishing diabetes mellitus from other diseases.

Previous works have reported that PAs are associated with aging, loss of skeletal muscle mass, and loss of fat-free mass (FFM)^33,43^, and that PAs are biomarkers of the skeletal muscle index, intracellular/extracellular water ratio index, and malnutrition state index^44–46^. In contrast, through an age-, BMI- and sex-matched study design, our results showed significantly lower PAs in diabetic patients than those in healthy controls, but we found no significant differences between these two groups in muscle mass, skeletal muscle mass, segmental muscle mass, intracellular water (ICW), extracellular water (ECW), total body water (TBW), and body cell mass; however, the ECW/TBW ratio was significantly different (see Table A3). These results imply that the lower PAs in diabetic patients are not consequences of changes in body composition, including muscle mass, skeletal muscle mass, and body cell mass, and are therefore biomarkers of diabetes independent of body composition parameters.

Some controversies have been reported in the literature regarding the performance of PAs for diagnosing diabetes. Buffa *et al*. reported that the whole-body PA was higher in diabetic patients than that in healthy controls based on a single-frequency impedance analysis (the specific frequency was not mentioned)^22^. On the other hand, two years later, Dittmar *et al*.^16^ reported that the whole-body PAs at 50 and 100 kHz were lower, but the PA at 5 kHz was higher in diabetic patients. An earlier work reported that the PA at 50 kHz was lower in people with diabetes^20^. In our study, we found that the segmental PAs at all measured frequencies and the PAmax in the subjects with diabetes were lower than those in the controls regardless of gender. For comparison with previous results, we calculated whole-body PAs and found that the whole-body PAs at 3 frequencies (5 kHz, 50 kHz, and 250 kHz) were lower in the diabetic patients than those in the controls. All these reports, including ours, are limited by small sample sizes. Different impedance devices and different ethnic groups are other possible reasons for the conflicting results. Multicenter studies with larger cohorts using a standardized device type/brand are needed to confirm our results.

Finding effective MF-BIA frequencies for diagnostic classification is another research topic. Historically, the single-frequency BIA method at 50 kHz was first adopted for clinical application, but this single-frequency BIA method was soon replaced by MF-BIA with additional frequencies such as 5 kHz, 100 kHz, 250 kHz, 500 kHz and 1 MHz in the late 1990 s^3,47–49^. However, these frequencies were based on an experiential and intuitional method with no solid theoretical background. To overcome uncertainty in frequency selection in commercial devices, we proposed a frequency-independent phase angle, “PAmax”, that is easily obtainable by a circular equation in a Cole-Cole model. The PAs and frequency selections obtained by this method are independent from device-specific frequency selections of the diverse range of BIA devices. More research must be conducted to determine frequencies that have clinical effectiveness and to formulate theoretical approaches utilizing existing commercial devices.

The number of significant differences in the segmental PAs between the two groups measured 30, 60, 90, and 120 minutes after meal intake became greater than those before meal intake. These results may be related to dyspepsia, an adverse effect observed in diabetic patients, because some papers reported dyspeptic symptoms^50^ in diabetes or gastric emptying and dysphagia in diabetic subjects^51^. Correlations between the PA depending on the time and flow of dyspeptic symptoms are worth studying in the future to improve the screening accuracy of diabetes. Some studies showed that the bioimpedance PAs could be a valuable diagnostic indicator in people with diabetes mellitus. The segmental PAs seem to be a useful indicator for the prevention and management of diabetes. However, further studies on the different mechanisms underlying the difference between the PAs of the diabetes patients and control subjects are required for use in clinical practice or as diagnostic equipment for diabetes. In particular, the discrepancies between the segmental PAs according to gender, reasons for reduced PAs in people with diabetes, and the increase in the significant differences for segmental PAs after a meal intake should be studied in further detail.

### Limitations

This study has some limitations. First, it was limited by its single-centered design, small sample size, and age criteria of enrolling subjects older than 40 years. The age-dependent trend of PAs in a broad population should be considered to increase the power of future studies^46^, and site-dependent systematic error should be cross-checked with a multicenter study design. Second, this was a cross-sectional study. A long-term follow-up study will reveal disease-period-dependent changes in the PAs and other BIA parameters.

## Methods

### Study subjects and experimental procedures

From June 2016 to February 2017, a total of 90 participants including 45 people with diabetes mellitus (26 males and 19 females) and 45 control subjects (26 males and 19 females) older than 40 years old were recruited at Dunsan Korean Medicine Hospital of Daejeon University. For the sampling strategy, subjects were sampled using Neyman’s allocation method and stratified by sex, age, which was divided into three strata (A1: 40–49 years, A2: 50–59 years, and A3: ≥60 years), and BMI, which was divided into two strata (B1: <25 kg/m^2^ and B2: ≥25 kg/m^2^). To obtain appropriate cell proportions for the allocation, we used information from a previous 2010 population and housing census from the South Korea and Korea National Health and Nutrition Examination Survey in 2014, which was provided by the Korean Statistical Information Service. This observational study was registered with the Clinical Research Information Service (CRIS) under the registration number, KCT0002132^52^. The study protocol was approved by the Institutional Review Board (IRB) of Dunsan Korean Medicine Hospital of Daejeon University (IRB number: DJDSKH-16-BM-04). Subjects with hypertension (a SBP higher than 160 mmHg or a DBP higher than 90 mmHg), severe renal disease, thyroid disease/hypothyroidism, liver dysfunction, cardiovascular disease, a pacemaker, hypersensitivity to electronic devices, anemia symptoms, physical handicaps and/or uncompensated chronic diseases were excluded. Pregnant women were also excluded. Patients with diabetes mellitus who were defined as having fasting hyperglycemia (a fasting plasma glucose (FPG) level of 126 mg/dl (7.0 mmol/l) or higher and less than 250 mg/dl (13.9 mmol/l), a FPG level higher than 250 mg/dl, or an HbA1c level higher than 48 mmol/mol (6.5%)) were excluded because of risk. Mild or moderate Type 1 or Type 2 diabetes mellitus patients whose blood glucose levels were controlled through meals or drugs according to the WHO criteria were included^53,54^. The FPG levels and HbA1c concentrations were measured from the blood plasma of a vein using standardized laboratory methods. Control subjects included those without a history of Type 2 diabetes mellitus, with a FPG level less than 100 mg/dl, and with an HbA1c level less than 42 mmol/mol (6%). All participants were informed of the objectives and methods of the research, and they provided written informed consent. The study was performed in accordance with Declaration of Helsinki guidelines. Bioimpedance parameters, including PAs and blood glucose levels, were measured 5 times, every 30 minutes starting before meal intake until 2 hours after meal intake. The PAs of the diabetic patients and controls were analyzed by a t-test, and correlations between the blood glucose levels and PAs were observed. The maximum reactance and PAs at the maximum reactance induced by a theoretical model were analyzed to increase the classification accuracy between the diabetic patients and controls.

### Multi-frequency bioimpedance measurement

**Figure 4:**
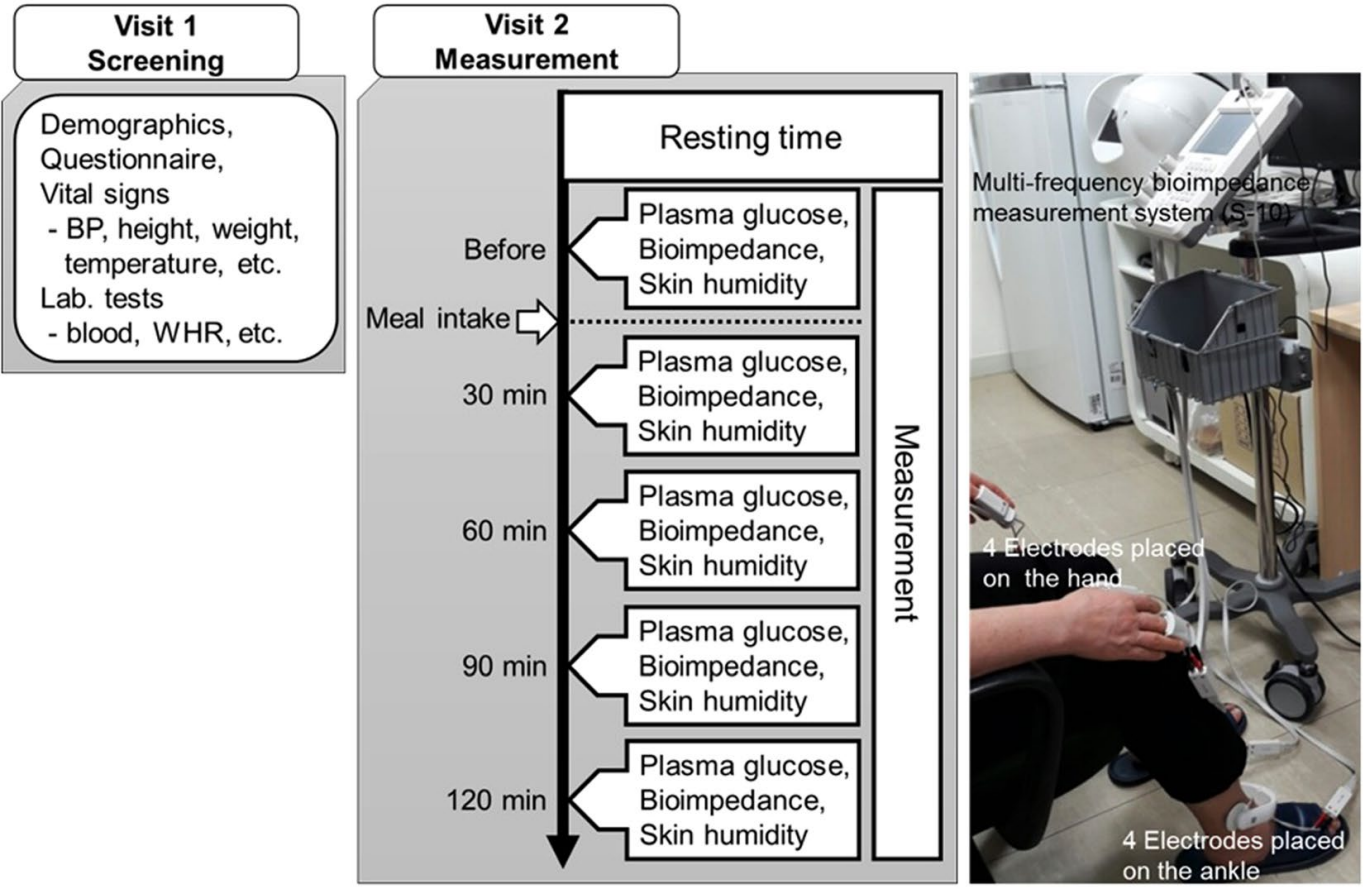
Measurement procedure and image of a subject during segmental PA measurements using a multi-frequency bioimpedance measurement system.

Bioelectrical impedance was measured using a direct segmental multi-frequency bioelectrical impedance analyzer with tetrapolar 8-point electrodes (InBody S10, InBody, Korea). We measured the impedance at six frequencies, 1, 5, 50, 250, 500, and 1000 kHz, and reactance and PAs at three frequencies, 5, 50, and 250 kHz. Eight electrodes were used to measure five segmental impedances of the body. Four were in contact with the thumb and index fingers of each hand, and four were in contact with the interior and exterior sides of each ankle. The plasma glucose level and bioimpedance of each participant were measured 5 times before and every 30 minutes after meal intake in the morning. The participants in this clinical trial fasted for over 9 hours overnight, abstained from alcohol, and avoided intense physical activity for 24 hours before the measurements. After being sufficiently stabilized, bioimpedance measurements were performed on the limbs of the body with the subjects in a sitting position. Figure 4 shows a schematic diagram of the measurement procedure and an image of measuring the segmental bioimpedance of a subject.

### Statistical analysis

The quantitative data were summarized as the mean and standard deviation (SD). Before performing the analyses, the extreme outliers in the measurement data were excluded by the 3*IQR (interquartile range) method. The 3*IQR method excludes data that are lower than Q_1_ − 3 × IQR or higher than Q_3_ + 3 × IQR, where Q_1_ and Q_3_ are the 25^th^ and 75^th^ percentiles, respectively. An independent two-sample t-test was applied to assess the mean difference between the diabetic patients and controls. For all analyses, the significance level was set to 0.05. Since we planned to test all of the bioimpedance measurements from each of the segments and frequencies, the original P-values were adjusted using the Benjamini-Hochberg procedure to reduce the family-wise error due to multiple tests for each measurement from the bioimpedance analyzer. In addition, Pearson’s correlation coefficients were also calculated to investigate the relationship between the PAs and direct or indirect indicators for diabetes such as glucose and HbA1C levels, the waist-to-hip ratio, and BMI. For further analysis, we calculated the areas under the curves (AUCs) of the receiver operating characteristics (ROC) curves for the PAs from each segment and frequency to ensure their capability as a diagnostic marker for diabetes.

## Conclusions

Using on a MTT for 45 diabetic patients and 45 control subjects who were matched for age, sex and BMI, we tested the ability of a MF-BIA method to noninvasively screening for diabetes mellitus. The segmental PAs at 50 kHz and 250 kHz in the diabetic patients were significantly lower than those in the normal controls. The segmental PAs were not influenced by the blood glucose level nor by the measurement time. In particular, the differences between the PAs of the diabetic patients and normal controls were most significant in the legs for females and in the arms for males. The proposed segmental multi-frequency PAs were shown to be good screening indicators for diabetes, as the AUC was 0.788 with the PA at 250 kHz in the LA for males, and the AUC was 0.845 with the PA at 250 kHz in the RL for females. We introduced “PAmax”, defined as the PA at the maximum reactance value, which can be obtained by the Cole-Cole model and is insensitive to the measurement frequencies of various multi-frequency BIA devices. As we showed that PAmax alone performed with a good classification accuracy (AUC = 0.827 for males and AUC = 0.803 for females), the PAmax would be useful biomarker for screening diabetes mellitus. To develop equipment for classifying or monitoring diabetes via segmental PAs, larger clinical studies are required.

## Electronic supplementary material

Appendix

## References

[1] Jackson AS, Pollock ML, Graves JE, Mahar MT (1988). Reliability and validity of bioelectrical impedance in determining body composition. J. Appl. Physiol, (1985).

[2] Abu Khaled M (1988). Electrical impedance in assessing human body composition: the BIA method. Am. J. Clin. Nutr..

[3] Shafer KJ, Siders WA, Johnson LK, Lukaski HC (2009). Validity of segmental multiple-frequency bioelectrical impedance analysis to estimate body composition of adults across a range of body mass indexes. Nutrition.

[4] Bosy-Westphal A (2006). Phase angle from bioelectrical impedance analysis: population reference values by age, sex, and body mass index. JPEN J. Parenter. Enteral. Nutr..

[5] Gupta D (2004). Bioelectrical impedance phase angle as a prognostic indicator in advanced pancreatic cancer. Br. J. Nutr..

[6] Kyle UG, Genton L, Pichard C (2013). Low phase angle determined by bioelectrical impedance analysis is associated with malnutrition and nutritional risk at hospital admission. Clin. Nutr..

[7] Ott M (1995). Bioelectrical impedance analysis as a predictor of survival in patients with human immunodeficiency virus infection. J. Acq. Immune De. Synd. Human Retrovir..

[8] Schwenk A (2000). Phase angle from bioelectrical impedance analysis remains an independent predictive marker in HIV-infected patients in the era of highly active antiretroviral treatment. Am. J. Clin. Nutr..

[9] Valdespino-Trejo A (2013). Low albumin levels and high impedance ratio as risk factors for worsening kidney function during hospitalization of decompensated heart failure patients. Exp. Clin. Cardiol..

[10] Martinez LC (2007). Bioelectrical impedance and strength measurements in patients with heart failure: comparison with functional class. Nutrition.

[11] Itobi E, Stroud M, Elia M (2006). Impact of oedema on recovery after major abdominal surgery and potential value of multifrequency bioimpedance measurements. Br. J. Surg..

[12] Mulasi U, Kuchnia AJ, Cole AJ, Earthman CP (2015). Bioimpedance at the bedside: current applications, limitations, and opportunities. Nutr. Clin. Pract..

[13] Kyle UG, Soundar EP, Genton L, Pichard C (2012). Can phase angle determined by bioelectrical impedance analysis assess nutritional risk? A comparison between healthy and hospitalized subjects. Clin. Nutr..

[14] Genton L (2017). Bioimpedance-derived phase angle and mortality among older people. Rejuv. Res..

[15] Kuchnia AJ (2017). Phase angle and impedance ratio: Reference cut-points from the United States National Health and Nutrition Examination Survey 1999–2004 from bioimpedance spectroscopy data. JPEN J. Parenter. Enteral. Nutr..

[16] Dittmar M, Reber H, Kahaly GJ (2015). Bioimpedance phase angle indicates catabolism in Type 2 diabetes. Diabet. Med..

[17] Beberashvili I (2014). Longitudinal changes in bioimpedance phase angle reflect inverse changes in serum IL-6 levels in maintenance hemodialysis patients. Nutrition.

[18] Colin-Ramirez E (2012). Bioelectrical impedance phase angle as a prognostic marker in chronic heart failure. Nutrition.

[19] Selberg O, Selberg D (2002). Norms and correlates of bioimpedance phase angle in healthy human subjects, hospitalized patients, and patients with liver cirrhosis. Eur. J. Appl. Physiol..

[20] Buscemi S, Blunda G, Maneri R, Verga S (1998). Bioelectrical characteristics of type 1 and type 2 diabetic subjects with reference to body water compartments. Acta Diabetol..

[21] Fein PA (2002). Usefulness of bioelectrical impedance analysis in monitoring nutrition status and survival of peritoneal dialysis patients. Adv. Perit. Dial..

[22] Buffa R (2013). Elderly subjects with type 2 diabetes show altered tissue electrical properties. Nutrition.

[23] Cuevas MAE (2010). Body fluid volume and nutritional status in hemodialysis: vector bioelectric impedance analysis. Clin. Nephrol..

[24] Pillon L, Piccoli A, Lowrie EG, Lazarus JM, Chertow GM (2004). Vector length as a proxy for the adequacy of ultrafiltration in hemodialysis. Kidney Int..

[25] Di Mauro M, Lazzarini D, Fumelli P, Carle F, Kosmidis A (2007). Bioelectrical impedance analysis and diabetes mellitus: which correlation among fructosamine, glycosylated haemoglobin and exchangeable potassium. Minerva Med..

[26] Ritz P, Salle A, Audran M, Rohmer V (2007). Comparison of different methods to assess body composition of weight loss in obese and diabetic patients. Diabetes Res. Clin. Pract..

[27] Wallymahmed ME, Morgan C, Gill GV, MacFarlane IA (2007). Aerobic fitness and hand grip strength in Type 1 diabetes: relationship to glycaemic control and body composition. Diabet. Med..

[28] Olive JL, Ballard KD, Miller JJ (2008). & Milliner, B. A. Metabolic rate and vascular function are reduced in women with a family history of type 2 diabetes mellitus. Metabolism.

[29] Prado-Olivarez J (2015). Bioimpedance phase angle analysis of foot skin in diabetic patients: an experimental case study. IRBM.

[30] Dreyer M (2011). Peripheral artery disease and disorders of microcirculation in patients with diabetes mellitus. Internist (Berl).

[31] Younes NA, Ahmad AT (2006). Diabetic foot disease. Endocr. Pract..

[32] Gorniak SL, Khan A, Ochoa N, Sharma MD, Phan CL (2014). Detecting subtle fingertip sensory and motor dysfunction in adults with type II diabetes. Exp. Brain Res..

[33] Yamada Y (2017). Electrical properties assessed by bioelectrical impedance spectroscopy as biomarkers of age-related loss of skeletal muscle quantity and quality. J. Gerontol. A Biol. Sci. Med. Sci..

[34] Johannsen L (2001). Evaluation of patients with symptoms suggestive of chronic polyneuropathy. J. Clin. Neuromuscul. Dis..

[35] Brem H, Tomic-Canic M (2007). Cellular and molecular basis of wound healing in diabetes. J. Clin. Invest..

[36] Pierson RN, Wang J (1988). Body composition denominators for measurements of metabolism: what measurements can be believed?. Mayo Clin. Proc..

[37] Brizzolara A, Barbieri M, Adezati L, Viviani G (1996). Water distribution in insulin-dependent diabetes mellitus in various states of metabolic control. Euro. J. Endocrinol..

[38] de Blasio F (2017). Raw BIA variables are predictors of muscle strength in patients with chronic obstructive pulmonary disease. Eur. J. Clin. Nutr..

[39] Araujo Antunes A (2012). Nutritional assessment of hospitalized HIV-infected patients by the phase angle z-score measurement. Nutr. Hosp..

[40] Norman K, Wirth R, Neubauer M, Eckardt R, Stobaus N (2015). The bioimpedance phase angle predicts low muscle strength, impaired quality of life, and increased mortality in old patients with cancer. J. Am. Med. Dir. Assoc..

[41] Marra M (2009). Bioelectrical impedance phase angle in constitutionally lean females, ballet dancers and patients with anorexia nervosa. Eur. J. Clin. Nutr..

[42] Ringaitiene, D. *et al*. Concordance of the new ESPEN criteria with low phase angle in defining early stages of malnutrition in cardiac surgery. *Clin. Nutr*, 10.1016/j.clnu.2017.08.007 (2017). 10.1016/j.clnu.2017.08.007 28843445

[43] Gonzalez MC, Barbosa-Silva TG, Bielemann RM, Gallagher D, Heymsfield SB (2016). Phase angle and its determinants in healthy subjects: influence of body composition. Am. J. Clin. Nutr..

[44] Marini E (2012). The potential of classic and specific bioelectrical impedance vector analysis for the assessment of sarcopenia and sarcopenic obesity. Clin. Interv. Aging.

[45] Dos Santos L, Cyrino ES, Antunes M, Santos DA, Sardinha LB (2016). Changes in phase angle and body composition induced by resistance training in older women. Eur. J. Clin. Nutr..

[46] Barbosa-Silva MC, Barros AJ, Wang J, Heymsfield SB, Pierson RN (2005). Bioelectrical impedance analysis: population reference values for phase angle by age and sex. Am. J. Clin. Nutr..

[47] Olde Rikkert MG, Deurenberg P, Jansen RW, van’t Hof MA, Hoefnagels WH (1997). Validation of multifrequency bioelectrical impedance analysis in monitoring fluid balance in healthy elderly subjects. J. Gerontol. A Biol. Sci. Med. Sci..

[48] Hills A, Byrne N (1998). Bioelectrical impedance and body composition assessment. Malays. J. Nutr..

[49] Yamada Y (2013). Comparison of single- or multifrequency bioelectrical impedance analysis and spectroscopy for assessment of appendicular skeletal muscle in the elderly. J. Appl. Physiol, (1985).

[50] Pfaffenbach B (1995). Antral myoelectric activity, gastric emptying, and dyspeptic symptoms in diabetics. Scand. J. Gastroenterol..

[51] Boltin D (2014). Vomiting and dysphagia predict delayed gastric emptying in diabetic and nondiabetic subjects. J. Diabetes Res..

[52] Ministry of Health and Welfare (Republic of Korea), KCT0002132, Comparison of human micro-current response and near infrared spectroscopy in diabetes and normal subjects Available from: https://cris.nih.go.kr/cris/search/search_result_st01_en.jsp?seq=6760&ltype=&rtype= (2016).

[53] World Health Organization. Definition and diagnosis of diabetes mellitus and intermediate hyperglycemia: report of a WHO/IDF consultation, http://apps.who.int/iris/bitstream/10665/43588/1/9241594934_eng.pdf (2006).

[54] World Health Organization. Use of glycated hemoglobin (HbA1c) in the diagnosis of diabetes mellitus, http://www.who.int/diabetes/publications/report-hba1c_2011.pdf (2011).

