# Supplementary material for: Glucose-independent segmental phase angles from multi-frequency bioimpedance analysis to discriminate diabetes mellitus
Source: Sci Rep. 2018 Jan 12;8:648. doi: 10.1038/s41598-017-18913-7 (PMC5766497; doi:10.1038/s41598-017-18913-7)
Supplement: Supplementary file 1 — Appendix [file 41598_2017_18913_MOESM1_ESM.pdf]

Paper title: Glucose-independent segmental phase angles from multi-frequency bioimpedance analysis to discriminate diabetes mellitus

Author list: Min-Ho Jun[1], Soochan Kim[2], Boncho Ku[1], JungHee Cho[1], Kahye Kim[1], Ho-Ryong Yoo[3], and Jaeuk U. Kim[1*]

Appendix

Table A1. Statistical comparison of the PAs in the right and left arms and legs

	Variable	No. (n=90)		Statistics
		Mean ± SD	Mean ± SD	P-values
Phase Angle (degrees)	5 kHz-RA_LA	2.53 ± 0.38	2.32 ± 0.37	< 0.001 ***
	5 kHz-RA_RL	2.53 ± 0.38	2.82 ± 0.65	< 0.001 ***
	5 kHz-LA_LL	2.32 ± 0.37	2.76 ± 0.61	< 0.001 ***
	5 kHz-RL_LL	2.82 ± 0.65	2.76 ± 0.61	0.449
	50 kHz-RA_LA	5.88 ± 0.72	5.45 ± 0.61	< 0.001 ***
	50 kHz-RA_RL	5.88 ± 0.72	6.74 ± 1.15	< 0.001 ***
	50 kHz-LA_LL	5.45 ± 0.61	6.52 ± 1.15	< 0.001 ***
	50 kHz-RL_LL	6.74 ± 1.15	6.52 ± 1.15	0.215
	250 kHz-RA_LA	5.96 ± 1.00	5.66 ± 0.97	0.041 *
	250 kHz-RA_RL	5.96 ± 1.00	3.73 ± 0.58	< 0.001 ***
	250 kHz-LA_LL	5.66 ± 0.97	3.69 ± 0.66	< 0.001 ***
	250 kHz-RL_LL	3.73 ± 0.58	3.69 ± 0.66	0.741

Table A2. Correlations between segmental PAs and disease duration

Variables	Phase angle vs. Duration			
	**Males**		**Females**	
	$r_{Pearson}$	***P-values***	$r_{Pearson}$	***P-values***
**5kHz-RA**	-0.313	0.120	-0.307	0.20073
**5kHz-LA**	-0.337	0.092	0.399	0.09063
**5kHz-RL**	-0.248	0.222	-0.537	0.01782

**5kHz-LL**	-0.300	0.137	-0.107	0.66403
**50kHz-RA**	-0.451	0.021	-0.463	0.04592
**50kHz-LA**	-0.377	0.058	-0.266	0.27044
**50kHz-RL**	-0.305	0.130	-0.580	0.00918
**50kHz-LL**	-0.347	0.082	-0.490	0.03331
**250kHz-RA**	-0.136	0.507	-0.474	0.04051
**250kHz-LA**	-0.116	0.571	-0.232	0.33916
**250kHz-RL**	-0.146	0.475	-0.459	0.04789
**250kHz-LL**	-0.205	0.316	-0.525	0.02093

Table A3. T-test results of body composition parameters provided by the InBody S10 device

Variables	Males (n=52)		Statistics	Females (n=38)		Statistics
	Diabetics (n=26) Mean ± SD	Controls (n=26) Mean ± SD	P-values	Diabetics (n=19) Mean ± SD	Controls (n=19) Mean ± SD	P-values
Muscle mass (kg)	52.25 ± 6.56	53.38 ± 6.28	0.528	39.13 ± 4.76	38.34 ± 4.77	0.615
Skeletal muscle mass (kg)	31.05 ± 4.24	32.01 ± 4.16	0.415	22.52 ± 2.95	22.27 ± 3.07	0.803
Muscle mass of RA (kg)	2.75 ± 0.42	2.85 ± 0.48	0.425	1.95 ± 0.35	1.82 ± 0.31	0.255
Muscle mass of LA (kg)	2.68 ± 0.46	2.79 ± 0.47	0.399	1.93 ± 0.35	1.79 ± 0.32	0.213
Muscle mass of TR (kg)	22.40 ± 2.73	23.15 ± 2.76	0.329	17.35 ± 2.19	16.71 ± 2.01	0.354
Muscle mass of RL (kg)	9.85 ± 1.37	9.93 ± 1.13	0.817	6.89 ± 0.94	6.84 ± 0.86	0.878
Muscle mass of LL (kg)	9.49 ± 1.34	9.68 ± 1.06	0.566	6.76 ± 0.95	6.70 ± 0.87	0.842
Intracellular water (ICW; l)	25.35 ± 3.25	26.08 ± 3.19	0.414	18.80 ± 2.26	18.61 ± 2.35	0.802
Extracellular water (ECW; l)	15.28 ± 1.85	15.35 ± 1.66	0.892	11.70 ± 1.50	11.20 ± 1.35	0.285
Total body water (TBW; l)	40.63 ± 5.07	41.43 ± 4.82	0.562	30.50 ± 3.73	29.81 ± 3.68	0.569
ECW/TBW (%)	37.64 ± 0.58	37.09 ± 0.73	0.004 **	38.34 ± 0.68	37.59 ± 0.70	0.002 **
Body cell mass (kg)	36.31 ± 4.65	37.36 ± 4.57	0.416	26.93 ± 3.24	26.66 ± 3.36	0.804

Table A4. Segmental phase angles with time and t-test results between diabetic patients and controls

	Segmental PAs (mean ± SD) with time (degrees)									
	Males (n=52)					Females (n=38)				
	Diabetics (n=26)<br>Controls (n=26)<br>*P*- values					Diabetics (n=19)<br>Controls (n=19)<br>*P*- values				
	Before MI	30 min.	60 min.	90 min.	120 min.	Before MI	30 min.	60 min.	90 min.	120 min.
**5 kHz-RA**	2.42 ± 0.31<br>2.66 ± 0.32	2.52 ± 0.33<br>2.79 ± 0.34	2.54 ± 0.34<br>2.88 ± 0.37	2.62 ± 0.40<br>2.92 ± 0.37	2.66 ±0.35<br>2.91 ± 0.39	2.12 ± 0.27<br>2.19 ± 0.25	2.29 ± 0.30<br>2.32 ± 0.35	2.19 ± 0.33<br>2.48 ± 0.30	2.31 ± 0.30<br>2.44 ± 0.32	2.34 ± 0.23<br>2.39 ± 0.23
	0.026 *	0.014 *	0.005 **	0.021 *	0.053	0.402	0.8	0.009 **	0.197	0.499
**5 kHz-LA**	2.24 ± 0.35<br>2.40 ± 0.25	2.36 ± 0.35<br>2.56 ± 0.32	2.36 ± 0.40<br>2.65 ± 0.34	2.40 ± 0.41<br>2.66 ± 0.38	2.40 ±0.39<br>2.64 ± 0.35	2.00 ± 0.27<br>2.01 ± 0.27	2.07 ± 0.29<br>2.14 ± 0.40	2.08 ± 0.32<br>2.17 ± 0.21	2.10 ± 0.30<br>2.22 ± 0.21	2.11 ± 0.30<br>2.25 ± 0.23
	0.159	0.097	0.017 *	0.048 *	0.036 *	0.906	0.495	0.313	0.158	0.115
**5 kHz-TR**	3.07 ± 1.10<br>2.96 ± 1.22	4.00 ± 1.95<br>3.05 ± 1.17	3.73 ± 0.82<br>3.17 ± 0.82	3.59 ± 2.06<br>3.37 ± 2.18	3.20 ±1.63<br>3.80 ± 3.89	2.73 ± 1.12<br>3.06 ± 1.39	3.07 ± 1.43<br>3.37 ± 1.44	3.47 ± 0.94<br>3.35 ± 1.04	3.38 ± 1.20<br>3.16 ± 1.14	3.23 ± 0.75<br>3.35 ± 0.75
	0.740	0.036 *	0.017 *	0.711	0.472	0.430	0.531	0.722	0.574	0.607
**5 kHz-RL**	2.98 ± 0.64<br>3.13 ± 0.57	2.86 ± 0.67<br>3.18 ± 0.64	2.88 ± 0.70<br>3.30 ± 0.55	2.93 ± 0.70<br>3.30 ± 0.55	2.99 ± 0.67<br>3.38 ± 0.66	2.26 ± 0.35<br>2.62 ± 0.50	2.21 ± 0.43<br>2.60 ± 0.40	2.18 ± 0.37<br>2.68 ± 0.49	2.28 ± 0.38<br>2.79 ± 0.53	2.26 ± 0.37<br>2.75 ± 0.53
	0.486	0.122	0.027 *	0.211	0.226	0.017 *	0.006 **	0.001 ***	0.002 **	0.002 **
**5 kHz-LL**	2.81 ± 0.57<br>3.00 ± 0.62	2.83 ± 0.67<br>3.04 ± 0.67	2.87 ± 0.70<br>3.16 ± 0.60	2.81 ± 0.53<br>3.22 ± 0.69	2.83 ±0.59<br>3.28 ± 0.75	2.23 ± 0.33<br>2.60 ± 0.58	2.24 ± 0.36<br>2.56 ± 0.45	2.22 ± 0.39<br>2.67 ± 0.48	2.23 ± 0.41<br>2.65 ± 0.49	2.27 ± 0.41<br>2.67 ± 0.50
	0.368	0.416	0.196	0.051	0.042 *	0.013 *	0.020 *	0.003 **	0.007 **	0.010 *
**50 kHz-RA**	5.89 ± 0.78<br>6.47 ± 0.58	5.78 ± 0.57<br>6.60 ± 0.81	5.78 ± 0.57<br>6.44 ± 0.55	5.97 ± 0.65<br>6.50 ± 0.60	5.97 ± 0.65<br>6.50 ± 0.56	5.24 ± 0.55<br>5.49 ± 0.50	5.43 ± 0.78<br>5.85 ± 1.18	5.34 ± 0.50<br>5.79 ± 0.58	5.35 ± 0.55<br>5.73 ± 0.74	5.44 ± 0.47<br>5.69 ± 0.83
	0.017 *	< 0.001 ***	0.005 **	0.018 *	0.018 *	0.147	0.431	0.020 *	0.081	0.573
**50 kHz-LA**	5.44 ± 0.62<br>6.01 ± 0.43	5.48 ± 0.56<br>6.09 ± 0.54	5.47 ± 0.60<br>6.11 ± 0.64	5.55 ± 0.58<br>6.19 ± 0.74	5.45 ± 0.58<br>6.05 ± 0.56	4.87 ± 0.51<br>5.14 ± 0.38	4.95 ± 0.51<br>5.24 ± 0.49	4.91 ± 0.50<br>5.24 ± 0.38	4.94 ± 0.54<br>5.18 ± 0.40	4.94 ± 0.50<br>5.22 ± 0.38
	0.002 **	0.002 **	0.002 **	0.003 **	< 0.001 ***	0.081	0.090	0.024 *	0.126	0.064
**50 kHz-TR**	6.43 ± 2.72<br>7.20 ± 2.80	7.17 ± 2.09<br>6.52 ± 2.84	7.45 ± 1.51<br>7.28 ± 2.06	6.45 ± 2.94<br>7.59 ± 3.84	5.88 ± 3.39<br>7.78 ± 4.55	5.71 ± 1.98<br>6.23 ± 1.66	5.16 ± 2.57<br>6.15 ± 1.51	6.27 ± 0.92<br>6.18 ± 2.48	6.00 ± 1.90<br>6.03 ± 2.18	5.61 ± 2.33<br>6.71 ± 1.22
	0.315	0.353	0.737	0.235	0.094	0.384	0.156	0.884	0.969	0.078
**50 kHz-RL**	6.98 ± 0.94<br>7.52 ± 0.91	6.86 ± 0.99<br>7.48 ± 0.97	6.75 ± 1.00<br>7.62 ± 1.01	6.79 ± 0.94<br>7.60 ± 0.99	6.83 ± 0.98<br>7.59 ± 1.03	5.76 ± 0.87<br>6.72 ± 1.06	5.62 ± 0.89<br>6.62 ± 0.94	5.58 ± 0.88<br>6.53 ± 0.94	5.54 ± 0.89<br>6.70 ± 1.06	5.51 ± 0.86<br>6.62 ± 1.07
	0.060	0.040 *	0.005 **	0.006 **	0.015 *	0.004 **	0.002 **	0.003 **	< 0.001 ***	0.001 ***
**50 kHz-LL**	6.69 ± 0.91<br>7.20 ± 1.21	6.55 ± 0.96<br>7.21 ± 1.26	6.50 ± 0.92<br>7.27 ± 1.28	6.51 ± 0.94<br>7.25 ± 1.25	6.54 ± 0.92<br>7.32 ± 1.34	5.73 ± 0.90<br>6.53 ± 1.05	5.66 ± 0.90<br>6.38 ± 0.86	5.51 ± 0.90<br>6.37 ± 0.90	5.53 ± 0.94<br>6.45 ± 0.99	5.56 ± 0.91<br>6.46 ± 1.04
	0.197	0.108	0.056	0.063	0.061	0.017 *	0.016 *	0.006 **	0.006 **	0.007 **
**250 kHz-RA**	6.02 ± 0.75<br>6.47 ± 0.61	5.76 ± 0.60<br>6.38 ± 0.55	5.67 ± 0.55<br>6.25 ± 0.57	5.77 ± 0.61<br>6.14 ± 0.51	5.71 ± 0.68<br>6.23 ± 0.66	5.47 ± 0.55<br>5.87 ± 0.52	5.67 ± 0.85<br>5.88 ± 0.63	5.41 ± 0.58<br>5.89 ± 0.58	5.39 ± 0.51<br>5.77 ± 0.59	5.29 ± 0.46<br>5.69 ± 0.60
	0.032 *	< 0.001 ***	< 0.001 ***	0.036 *	0.009 **	0.027 *	0.404	0.015 *	0.041 *	0.026 *
**250 kHz-LA**	5.75 ± 0.69<br>6.18 ± 0.47	5.49 ± 0.57<br>6.05 ± 0.47	5.47 ± 0.54<br>5.94 ± 0.46	5.52 ± 0.55<br>5.93 ± 0.51	5.38 ± 0.56<br>6.23 ± 0.66	5.18 ± 0.47<br>5.66 ± 0.53	5.23 ± 0.53<br>5.58 ± 0.56	5.11 ± 0.40<br>5.54 ± 0.51	5.12 ± 0.52<br>5.35 ± 0.41	5.01 ± 0.41<br>5.36 ± 0.40
	0.015 *	< 0.001 **	0.002 **	0.009 **	< 0.001 ***	0.005 **	0.062	0.006 **	0.129	0.010 **
**250 kHz-TR**	8.61 ± 2.75<br>9.36 ± 4.04	8.49 ± 2.66<br>9.02 ± 2.54	8.76 ± 1.50<br>8.74 ± 3.82	8.44 ± 2.49<br>8.38 ± 2.39	7.83 ± 2.93<br>9.46 ± 4.76	7.32 ± 2.02<br>7.97± 1.18	6.06 ± 2.94<br>7.28 ± 2.87	7.32 ± 2.02<br>7.69 ± 2.80	6.97 ± 3.13<br>7.38 ± 1.48	7.07 ± 2.31<br>8.02 ± 2.12
	0.438	0.464	0.977	0.928	0.144	0.232	0.204	0.400	0.608	0.194
**250 kHz-RL**	3.74 ± 0.55<br>4.28 ± 0.74	3.62 ± 0.54<br>4.02 ± 0.72	3.56 ± 0.47<br>3.96 ± 0..60	3.57 ± 0.46<br>3.98 ± 0.53	3.53 ± 0.54<br>4.06 ± 0.72	3.54 ± 0.64<br>4.38 ± 0.67	3.27 ± 0.50<br>4.04 ± 0.59	3.26 ± 0.65<br>3.77 ± 0.48	3.17 ± 0.55<br>3.88 ± 0.64	3.17 ± 0.51<br>3.92 ± 0.57
	0.010 **	0.040 *	0.011 *	0.008 **	0.009 **	< 0.001 ***	< 0.001 ***	0.009 **	< 0.001 ***	< 0.001 ***
**250 kHz-LL**	3.78 ± 0.54<br>4.28 ± 0.75	3.46 ± 0.48<br>4.12 ± 0.73	3.43 ± 0.57<br>4.16 ± 0.83	3.44 ± 0.58<br>4.12 ± 0.83	3.55 ± 0.58<br>4.11 ± 0.89	3.53 ± 0.83<br>4.08 ± 0.69	3.41 ± 0.69<br>3.85 ± 0.55	3.24 ± 0.71<br>3.74 ± 0.53	3.25 ± 0.70<br>3.79 ± 0.62	3.24 ± 0.65<br>3.82 ± 0.66
	0.024 *	0.003 **	0.003 **	0.005 **	0.036 *	0.033 *	0.036 *	0.019 *	0.015 *	0.011 *

MI: meal intake, 30 min.: 30 minutes after meal intake, 60 min.: 60 minutes after meal intake,

90 min.: 90 minutes after meal intake, 120 min.: 120 minutes after meal intake; P-values: * < 0.05, ** < 0.01, *** < 0.001
